# Active DNA end processing in micronuclei of ovarian cancer cells

**DOI:** 10.1186/s12885-018-4347-0

**Published:** 2018-04-16

**Authors:** Zizhi Tang, Juan Yang, Xin Wang, Ming Zeng, Jing Wang, Ao Wang, Mingcai Zhao, Liandi Guo, Cong Liu, Dehua Li, Jie Chen

**Affiliations:** 1 Department of Pharmacology, West China Second University Hospital, Key Laboratory of Birth Defects and Related Diseases of Women and Children (Ministry of Education), Sichuan University, Chengdu, 610041 People’s Republic of China; 2 Department of Gynecology and Obstetrics, West China Second University Hospital, Sichuan University, Chengdu, 610041 People’s Republic of China; 3 Department of Laboratory Medicine, Suining Central Hospital, 629000 Suining, People’s Republic of China; 4 College of Pharmacy, Southwest Minzu University, No.16 South Section 4, Yihuan Road, Chengdu, 610041 People’s Republic of China

**Keywords:** Micronucleus, DNA damage, Ovarian cancer, ssDNA

## Abstract

**Background:**

Ovarian cancer is one of the most deadly gynecological malignancies and inclined to recurrence and drug resistance. Previous studies showed that the tumorigenesis of ovarian cancers and their major histotypes are associated with genomic instability caused by defined sets of pathogenic mutations. In contrast, the mechanism that influences the development of drug resistance and disease recurrence is not well elucidated. Solid tumors are prone to chromosomal instability (CIN) and micronuclei formation (MN). Although MN is traditionally regarded as the outcome of genomic instability, recent investigation on its origin and final consequences reveal that the abnormal DNA metabolism in MN is a driver force for some types of catastrophic genomic rearrangements, accelerating dramatic genetic variation of cancer cells.

**Methods:**

We used Indirect Immunofluorescent staining to visualize micronuclei and activation of DNA repair factors in ovarian cancer cell lines and biopsies.

**Results:**

We show that ovarian cancer cells are disposed to form micronuclei upon genotoxic insults. Double strand DNA breaks (DSBs)-triggered insurgence of micronuclei is associated with unrepaired chromosomes passing through mitosis. According to their morphology and DNA staining, micronuclei compartments are divided into early and late stages that can be further characterized by differential staining of γH2AX and 53BP1. We also show that MN compartments do not halt controlled DNA metabolism as sequestered nuclear repair factors are enriched at DNA breaks in MN compartments and efficiently process DNA ends to generate single-stranded DNA (ssDNA) structures. Interestingly, unknown factors are required for DNA end processing in MN in addition to the nuclear resection machinery. Finally, these hallmarks of micronuclei evolution depicted in cell culture were recapitulated in different stages of ovarian cancer biopsies.

**Conclusions:**

In aggregate, our findings demonstrate that ovarian cancer cells are inclined to form micronuclei that undergo robust DNA metabolism and generate ssDNA structures, potentially destabilizing genomic structures and triggering genetic variation.

## Background

Ovarian cancer is an aggressive gynecological malignancy with approximately 30% of overall 5-year survival rate, seriously threatening women’s health. Epithelial ovarian cancer (EOC) accounts for the majority of ovarian malignancies and for 90% of advanced-stage disease and mortality. Although cytoreductive surgery combined with chemotherapy takes effect in initial treatment of EOC, current therapy is very limited due to the asymptomatic nature of the early stages and about 60% patients become resistant to chemotherapy [1]. Thus, EOC has both a poor prognosis and a high fatality rate.

A plethora of report suggests that loss of genome stability contributes to the tumorigenesis of EOC [2]. Major histotypes of EOC associate with genetic alterations and genomic instability: high-grade serous ovarian cancer (HGSC) present high-penetration of *TP53* and *BRCA1*/*BRCA2* mutations while other types of OC (clear cell and endometrioid cancer) harbor detrimental *ARID1A* mutation [3]. As pathogenic *BRCA1*/*BRCA2* mutations are highly mutagenic, causing large amount of SNV, copy number variation and structural variations and destabilizing the genome, a significant proportion of ovarian cancer display genomic and chromosomal instability attributed to DNA repair deficiencies. Partially due to the CIN caused by these pro-cancerous mutations, the post-therapy somatic genome of EOC is under quick evolution, displaying spatial and temporal heterogeneity in terms of genetic composition [4–6]. The heterogeneity in ovarian cancer tissue results in branched evolution that contributes to the arousal of drug resistance [7, 8].

The CIN of ovarian cancer is caused by defective DNA damage responses (DDR). DDR is activated upon genotoxic challenges to arrest cell-cycle and DNA replication, and triggers repair pathway to eliminate DNA lesions and preserve genome stability [9, 10]. The phosphorylation of H2AX on its Ser139, or γH2AX, is triggered in the very early phase of DNA damage and can form damage-induced foci in the chromatin regions of damaged DNA [11]. γH2AX sets up a platform by which damage recognition factors and repair molecules including MDC1, TopBP1, RAD9-RAD1-HUS1, MRE11, RAD50, NBS1 and TP53, are recruited to execute DNA repair [9, 12]. Repair factors like BRCA1 and 53BP1 regulate DSB repair by balancing the pathway choice between non-homologous end joining (NHEJ) and homologous recombination (HR) [13]. The concordant action of these proteins play crucial roles in controlling the DNA end resection at early stage of DSB repair, generating appropriate amount of ssDNA that is required for HR. Insufficient processing of DNA end results in decreased HR while excessive or prolonged presence of aberrant ssDNA structures are potentially toxic to cells ([14] and unpublished data). Genetic impairment of these signaling mechanism or challenging of the cancer cells by chemo- or radiotherapy causes radical mutagenic processes and confers quick evolution of cancer genomes.

In addition to genetic aberrance caused by impaired DDR function in the nuclear compartment, recent study also suggest that micronucleus, DNA fragments disintegrated from daughter nuclei during preceding mitosis, plays important roles in genetic evolution of solid cancer. To date, most solid tumors and pre-cancerous lesions are shown to display elevated MN frequency due to inherent CIN [15]. Traditionally, micronuclei is viewed as the outcome of unrepaired DNA breaks and can be induced with genotoxic agents, such as chemical reagents or irradiation [16]. The level of MN constitutes a biomarker for chromosomal instability (CIN) [17, 18], or to predict sensitivity and outcome of radiotherapy [19, 20]. However, recent progress in high-resolution microscope and genomic sequencing suggest that MN has improperly controlled DNA metabolism with truncated DDR signaling, which can trigger massive genomic rearrangements and drive quick evolution of cancer cells via genetic variation [21].

Despite these progress in understanding of the roles of micronuclei in genomic stability, aspects of DNA metabolism within MN compartments, such as processing of DNA ends and re-integration of micronuclei DNA back to the genome, is poorly characterized. In this work, we investigated the DNA metabolism of MN in genotoxic challenged ovarian cancer cells. We characterized the recruitment of DDR factors in IR-induced micronuclei, and show that damage signaling including the chromatin recruitment of γH2AX, 53BP1 and DNA end processing is still active in micronuclei, which drives the evolution of micronuclei from early to late stages. Interestingly, micronuclei display different DDR patterns from main nuclei compartments in terms of DSB-recruitment of repair factors and employing of DNA end processing enzymes. Furthermore, hallmarks of DDR signals were demonstrated in off-nuclear compartments of clinically derived ovarian cancer biopsies. Taken together, our findings reveal the active DNA metabolism in micronuclei, generating intermediate DNA structures that potentially induce genomic rearrangements and drive evolution of cancer cells.

## Methods

### Cell culture

Ovarian cancer cell lines (OVCAR-8 and SKOV-3) purchased from ATCC were maintained in DMEM (GIBCO) supplemented with 10% Fetal Bovine Serum (FBS, Hyclone) and 100 U/ml Penicillin and 100 mg/ml streptomycin (GIBCO). Enzymatic digestion (Trypsin, GIBCO, 25200–056) was used for cell passage. All cells were propagated at 37 °C in 5% CO2 incubator. Primary ovarian cancer cells were isolated from fresh tissue blocks by enzymatic digestion (Collagenase A, Roche, 10,103,578,001; Trypsin, GIBCO, 25200–056) for 2 h with occasional vortex. Isolated single cells were dispersed in RPMI-1640/10% FBS and grown in media supplemented with 10 mg/ml Metrigel (BD, 356234). After 48 h, experiments were performed when 70% of cells were attached.

### Sample collections from patient

Normal or ovarian cancer tissue samples were collected from West China Second University Hospital, Sichuan University. All performance of sample request, collection and processing were informed to and consented by patients, and were carried out in accordance with the Ethics Guidelines and Regulation of the West China Second University Hospital, Sichuan University. Biopsies from 10 patients were analyzed in this study and data from three cases were presented. All patients were provided with written documents and consented the use of their tissues along with the extraction of their routine histopathological diagnosis for research purpose.

All women were surgically treated at the unit of gynecological surgery at the West China Second University Hospital for different gynecological reasons. Five ovarian tissues diagnosed with ovarian cancer were included in this study along with one ‘healthy’” ovary without any detectable malignancy. All patients did not receive chemotherapy upon sample collection. Part of the ovarian cortex biopsy was sent for routine pathological investigations, and another part was collected for experiments in this study.

### Cryosections from biopsies of ovarian cancer

For histological staining, freshly excised tissue blocks were embedded in OCT, followed by snap frozen in liquid nitrogen and stored in -80 °C. Deep frozen tissue blocks were sectioned at 8 μm using microtome (LEICA, CM3050). For ex vivo culture of ovarian tissue, biopsies were cut into small pieces of 1–2 mm^3^, followed by digestion and cell culture procedure as described as above.

### Irradiation and chemical treatment

Cytotoxic chemical or inhibitors were added to cell culture for indicated period of time for respective assays and at following concentrations: Cisplatin (4 μg/ml; Supertrack Bio-pharmaceutical, 131,102), MMS (0.5 mg/ml); Camptothecin (2 uM; Selleck, S2423), Mitomycin C (0.2μg/ml; Selleck, S8146), Paclitaxel (35 uM; Selleck, S1150), Nocodazole (100 ng/ml; Sigma, M1404) and hydroxyurea (5 mM; Selleck, S1896); Caffeine (20 μg/ml; Sigma, 58-08-2). Ionizing radiation was performed at 1 Gray/min using custom-made X-ray machine (Wandong Ltd., Beijing) and dosages were described in respective experiments.

### Immunostaining and fluorescence microscopy

Cells grown on cover slips were fixed with 4% paraformaldehyde (PFA), and permeabilized in 0.3% Triton X-100. Cells were blocked at 37 °C for 30 min with 3% BSA in PBS supplemented with 3% donkey serum and 0.2% Triton X-100. Fixed cells were then incubated with diluted primary antibodies: Rabbit anti-53BP1 (Cell Signaling, 3428P), Mouse anti-γH2AX (Millipore, 05–636), Rabbit anti-NBS1-pSerine 343 (Epitomics, 2194–1-1), BRCA1-pS1524 (Bethyl, A300-001A), Mouse anti-TP53-pSerine 15 (Cell Signaling, 2524); Rabbit anti-RPA32-pSerine 33 (NOVUS, NB100–544), Rabbit anti-ssDNA (IBL,18731). After extensive wash by PBS, coverslips were incubated with secondary antibodies (Rabbit IgG F(ab′)_2_ fragment-Cy3 and goat anti-Mouse IgG-FITC antibody, Sigma) for 30 min. After mounting with DAPI solution (VECTOR, H-1200), images were obtained using an Olympus epifluorescent microscope (BX 51) and analyzed with Image-pro plus (Applied Imaging). 100–200 cells or MN from 3 independent experiments were counted for quantitative immunofluorescent assays.

### Statistical analysis

Statistical analysis was performed with SPSS 20.0 software. All data was from three parallel assays. Student’s *t*-test (two-tailed) and one-way ANOVA was the basis of statistical significance calculating and the level of significance was set at *P* < 0.05. Correlation was calculated by using Pearson’s correlation equations. Microsoft Office Excel 2003 was used for plotting.

## Results

### Tendency of micronuclei formation in ovarian cancer cells

**Fig. 1:**
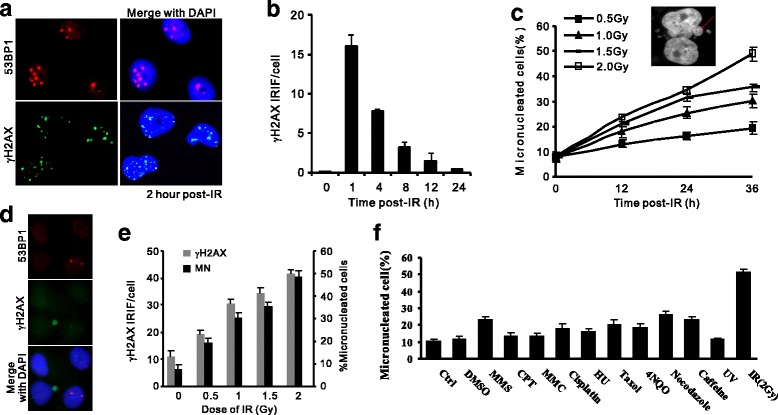
Genotoxicity-induced micronuclei in ovarian cancer cells. **a**: Induction of DSBs in OVCAR-8 cells after 1 Gy ionizing radiation. γH2AX and 53BP1 foci and nuclei morphology were monitored by indirect immunofluorescent staining (IF) at indicated time post-IR. Nuclei were indicated by DAPI staining. **b**: Dynamics of γH2AX IRIF in OVCAR-8 cells after 1 Gy irradiation. *n* = 3 biological repeats. Error bars = s.d. **c**: Time and dosage-dependent increase of MN IR treatment according to DAPI staining. Inset: micronuclei (red arrows) visualized by DAPI staining. **d**: γH2AX and 53BP1 staining by IF for OVCAR-8 12 h post-IR, showing unrepaired DSBs in both MN and nuclei compartments. **e**: Counting of γH2AX (Left vertical axis) and percentage of cells containing MN (Right vertical axis) at indicated dosages of IR irradiation. **f**: Induction of MN upon various genotoxic treatments. Percentage of micronucleated cells was enumerated according to DAPI staining. Apparently, OVCAR-8 is more liable to MN formation after ionizing radiation in comparison to other treatments. Cytotoxic chemicals were treated for 4 h and their concentrations are described in Methods. UV: 5 J/m^2^; IR: 2 Gy

To characterize micronuclei formation in ovarian cancer cells, we induced DSBs by ionizing irradiation (IR) in an OC cell line (OVCAR-8). One hour after IR treatment, plenty of γH2AX and 53BP1 ionizing radiation-induced foci (IRIF) representing DSBs emerged (fig. 1a). γH2AX is recruited to DSB after IR exposure and decline when damages are eliminated (fig. 1b). Twelve hours later, while γH2AX IRIF diminished, increment of micronuclei was observed, showing a dosage-dependent effect (fig. 1c). In these IR-induced micronuclei compartments, a significant fraction contained DSBs marked by γH2AX foci, consistent to previous works reports showing that micronuclei contains broken chromosomes expel from main nuclei [16, 22] (fig. 1d). The micronuclei induction is due to the generation of DSBs as enumeration of IR-induced MN at 12 h post-IR was well correlated with the number of γH2AX IRIF immediately after irradiation (fig. 1e). To evaluate the MN induction by different genotoxic reagents, OVCAR-8 cells were subjected to treatments of replication inhibitor (Hydroxyurea/HU, Caffeine), clastogens (Camptothecin/CPT, Mitomycin C/MMC, Methyl methanesulfonate/MMS, Cisplatin), UV radiation mimic (4-NQO) and radiation (Ultraviolet/UV and IR) (fig. 1f). Strikingly, although all reagents but UV induced MN at different levels, IR treatment displayed the greatest potential to induce micronuclei compared with other mutagens. Thus, OVCAR-8 cells are inclined to micronuclei formation after genotoxic challenges, especially upon DSB generation.

### Abnormal mitosis associated with increment of micronuclei

**Fig. 2:**
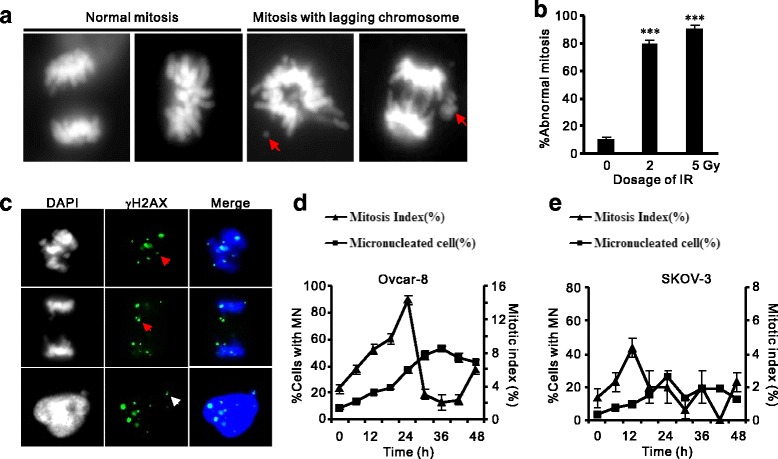
IR-induced micronuclei in OVCAR-8 cells are associated with aberrant mitosis. **a**-**b**: Representative images (**a**) and quantification (**b**) of abnormal mitoses in post-IR OVCAR-8 cells. Normal and aberrant mitosis were visualized by DAPI. Red arrows: lagging or broken chromosomes. *n* = 3 biological repeats. Error bars = s.d. ***P < 0.001 (*t*-test). **c**: Localization of γH2AX associated with broken chromosomes of mitotic cells (red arrows) or MN aside post-mitotic nucleus (white arrows). **d**-**e**: Enumeration of MN formation (Left vertical axis) and mitotic index (Right vertical axis) and in OVCAR-8 (**d**) and SKOV-3 (**e**) cells at 12 intervals after IR treatment (2 Gy). Both cell lines showed increased post-IR MN formation correlated with earlier changes mitotic index

Abnormal mitosis emerges in OVCAR-8 cells after radiation exposure, occurring as lagging chromosomes, chromosomal bridges or severed DNA fragments (fig. 2a). Prevalent aberrant mitosis were detected in irradiated OVCAR-8 cells as visualized by DAPI (4′,6-diamidino-2-phenylindole) staining, with 79.3% and 90% after 2 Gy and 5 Gy radiation in comparison with untreated (9.6%, fig. 2b), reminiscent of the elevated level of MN with increased IR dosage (fig. 1c). These abnormal mitoses were frequently associated with damaged DNA as γH2AX IRIF on condensed telophase chromosomes was observed in lagging chromosomes, chromosomal bridges, and in extruded post-mitotic micronuclei (fig. 2c), indicating that chromosomes containing unrepaired DSBs accompany the entry of mitosis and are liable to form off-nuclei MN in the coming interphase.

We monitored the correlation of MN formation and mitotic index (MI) in OVCAR-8 and another OC cell line (SKOV-3) in a longer time span of cell cycle after IR treatment. MI of OVCAR-8 cells culminated at 24-h after irradiation (14%) and declined till 36 h post-IR (fig. 2d). Strikingly, micronuclei emerged in these cells followed the wave of mitosis, peaked at 36 h and lagged of MI climax for about 12 h. Similarly, the association of mitosis and micronuclei induction was reproduced in a second ovarian cancer cells (SKOV-3), though the induction of micronuclei is less pronounced than OVCAR-8 (fig. 2e). Therefore, frequent chromosomal aberrance and passage of unrepaired DSBs through mitosis contribute to the formation of micronuclei in ovarian cancer cells.

### Evolution of cytoplasmic micronuclei

**Fig. 3:**
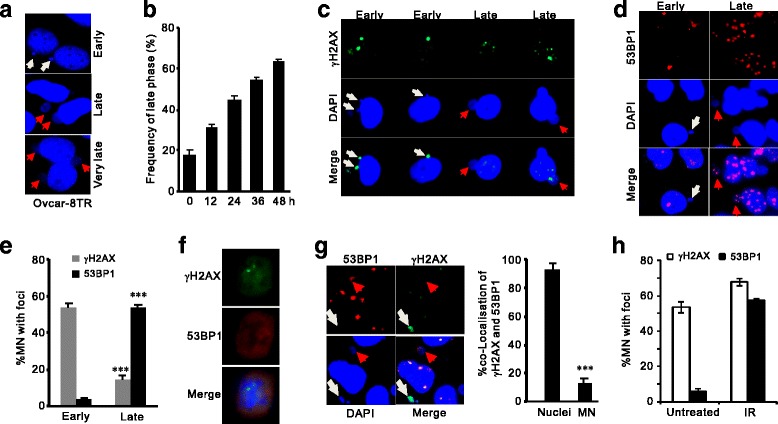
Differential DDR markers in early and late phases of micronuclei. **a**: DAPI staining showing the dynamic changes of IR-induced MN in OVCAR-8.Early MN (white arrows) exhibited dense and compact DAPI staining while turned into loose structures with weaken DAPI staining upon transforming into late stages (red arrows). **b**: Counting for late stage MN after 2-Gy irradiation at indicated time points. n = 3 biological repeats. Error bars = s.d. **c**-**e**: Indirect immunofluorescent staining for γH2AX (**c**) or 53BP1 (**d**) IRIF and quantifications (**e**) in early and late phases of MN. OVACAR-8TR cells were fixed and stained 36 h after IR treatments. Note that the lack of γH2AX foci and enriched 53BP1 foci in late stages. White arrows: early-stage MN; Red arrows: late-stage MN. **f**: IF staining of γH2AX and 53BP1 foci in post-IR mitotic cells, showing the retaining of γH2AX foci and exclusion of 53BP1 signal from chromosomes. **g**: Lack of γH2AX and 53BP1 co-localization in micronuclei compartments. Left: representative images showing strong γH2AX signals in early stages and 53BP1 foci in late stages. Right: calculation for percentage of γH2AX/53BP1 double positive foci in nuclear and MN compartments. Majority of these foci were overlapping in nuclei while co-localization events were rare in MN. n = 3 biological repeats. Error bars = s.d. ***P < 0.001 (*t*-test). Hh: Counting of γH2AX and 53BP1 foci in untreated (spontaneous) and IR-induced MN

Micronuclei are still active in DNA metabolism reflecting in their dynamic morphology [23]. We firstly used DAPI staining to visualize micronuclei at different time points. Apparently, most micronuclei detected within 12 h were small and compact, easily staining and captured by DAPI staining (fig. 3a). In contrast, a significant proportion of micronuclei in later post-IR windows (36 h) displayed larger volume and more resistant to DAPI staining. Accordingly, we categorize micronuclei into three stages: early-phase of compact with DAPI staining, late-phase of enlarged volume but visible DAPI signals, and very late phase MN with very weak DAPI signals that can only be captured by imaging enhancement (fig. 3a). The proportions of MN at different stages changed after IR: the late and very late stages accounted for 17.7% of total MN before IR treatment while increased to 63% at 48 h post-IR (fig. 3b). Thus, dynamic changes of morphology in micronuclei reflect a gradual evolution of MN with correlated changed of DNA status.

### DSB recruitment of signaling factors in micronuclei is different from nuclear DDR

The off-nuclei micronuclei compartments still contain the nucleoplasmic components that are necessary for DNA metabolism. Enclosed repair factors, like γH2AX and 53BP1, are liable to be attracted to DNA breaks in post-mitotic micronuclei. We monitored the activation of these DSB factors in different MN compartments. As shown in Fig. 3c, γH2AX was predominantly positive in early micronuclei (53.5%) while diminished in late-stage compartments (14.2%). On the contrary, 53BP1 displayed opposite distribution pattern as early-stage micronuclei hardly contained 53BP1 foci but exhibited a dramatic enrichment in late stages (3.4% versus 53.9%) (figs. 3d and e). Lack of 53BP1 in early MN could be explained by the exclusion of 53BP1 from the mitotic DSBs where γH2AX loading were well preserved (fig. 3f). This mechanism results in immediate appearance of γH2AX IRIF in post-mitotic MN, whereas 53BP1 only aggregates to DSBs in late-stage compartments. As such, in contrast to the DSB loading of repair factors in main nuclei bodies, co-localization of γH2AX and 53BP1 foci in IR-induced micronuclei compartments was rare (Nuclear compartments: 92.5%, MN compartments: 13.2%; fig. 3g). Thus, although nuclear repair factors participate in DDR in micronuclei, the signaling mechanisms are different between these separated compartments.

Moreover, we noticed the differential DSB-recruitment of γH2AX and 53BP1 in spontaneous (without irradiation) and DSB-induced MN. The number of γH2AX was comparable (53.5% and 67.7% in spontaneous and post-IR MN, respectively), whereas spontaneous MN containing 53BP1 foci was rare, in sharp contrast to the substantial accumulation of 53P1 foci in IR-induced compartments (5.7% versus 57.3%, fig. 3h). This lack of 53BP1 foci in spontaneous MN may reflect its origin from replication stress, where accumulation of ssDNA instead of DSBs promotes the phosphorylation of H2AX but excludes loading of 53BP1. Thus, spontaneous micronuclei in OVCAR-8 cells are mainly stemmed from chromosomal fragments containing replicational damages whose damage signaling is distinct from DSB-induced MN.

### Extensive DNA end processing in late-stage micronuclei

**Fig. 4:**
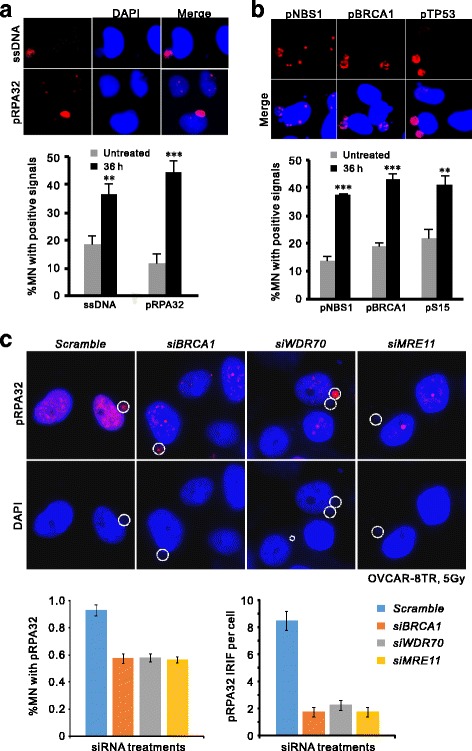
Active DNA end processing in late micronuclei compartments. **a**: Detection and quantification of RPA32 phosphorylation (pSerine 33) and ssDNA by specific antibodies in MN compartments of OVCAR-8 cells 36 h after 2-Gy irradiation. Note the nuclear signals have reduced while those in MN were intense. n = 3 biological repeats. Error bars = s.d. **P < 0.01; ***P < 0.005 (*t*-test). **b**: Immunostaining and enumeration of phosphorylated NBS1-pSerine 343, BRCA1-pSerine 1524 and TP53-pSerine 15 in MN compartments. **P < 0.01; ***P < 0.005 (*t*-test). **c**: Representative images (Upper panel) and quantification of RPA signal in MN compartments (Lower left) or RPA foci in nuclei (lower right) of post-IR OVCAR-8 cells after indicated treatments. Pro-resection genes (MRE11, BRCA1 and WDR70) were knockdown by specific siRNA. Percentage of positive RPA32-pS33 MN population was calculated after 36 h of IR treatment

The changes of DAPI staining in early and late stage MN suggest alteration of DNA status: while DAPI fluorescent dye binds to the major groves of intact double strand DNA instead of modified DNA structure like single stranded DNA (ssDNA) resected from DSB ends, the attenuated affinity of DAPI for DNA content in late-stage MN suggests collapse of double stand DNA, possibly caused by nucleases that process broken DNA ends. We were prompted to examine the activity of DNA end resection that may cause the disappearance of DAPI signals in late stages of micronuclei. We visualized ssDNA by immunostaining with antibodies specific to ssDNA and phosphorylated replication protein A (RPA32) that coats the ssDNA filaments [24]. Micronuclei displayed a significant increase of RPA32 phosphorylation at 36 h after IR in comparison to untreated cells (44.7% vs. 11.7%), and so as to the ssDNA signal per se (36.7% vs. 18.3%, fig. 4a). Moreover, generation of ssDNA accompanied with the activation of repair factors indicated by the phosphorylation of NBS1, BRCA1 and TP53 (Fig. 4b). Conspicuously, all of these DDR factors were mainly detected in late-phase MN with weak DAPI staining, while the corresponding signals have diminished in main nuclear compartments (fig. 4a and b), indicating slow ssDNA processing kinetics in MN. Intriguingly, although knocking down of a set of ssDNA processing factors including BRCA1, MRE11 and WDR70 (an epigenetic regulator for long-range resection) [25, 26] dramatically inhibited the formation of IR-induced RPA foci in main nuclei, RPA32 phosphorylation in MN was only marginally affected (fig. 4c), indicating ssDNA processing is not synchronized between MN and nuclei and likely involved in distinct resection factors. Thus, we conclude that DNA lesions in MN are subjected to structural processing that generates a significant amount of ssDNA via partially overlapped mechanism of nuclei.

### Micronuclei formation in ovarian cancer

**Fig. 5:**
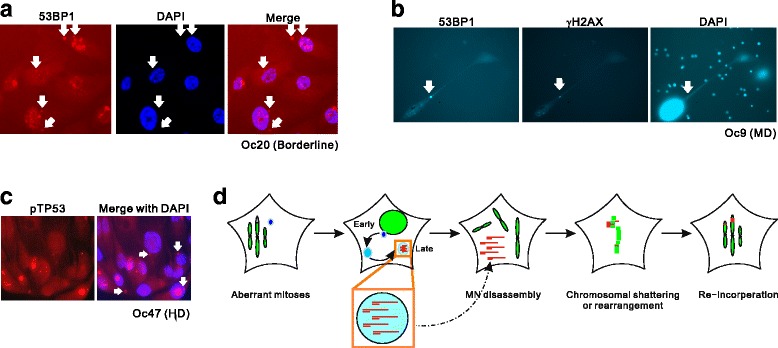
Detection of micronuclei in biopsies of human ovarian cancer. **a**: Micronuclei-like structure (white arrows) labeled with 53BP1 in primary culture isolated from OC47 tissue (borderline serous cancer). DAPI staining of these cytoplasmic structures were invisible. **b**: Micronuclei (white arrows) marked with 53BP1 and γH2AX in primary culture from OC9. Note although the image capture for DAPI staining was over-exposed, it was still invisible at the position of 53BP1/γH2AX-positive compartment (white arrow). OC9: moderately differentiated serous cancer. **c**: Detection of MN-like structures in cryosections of human ovarian cancer double labeled with DAPI and phosphorylated TP53-pSerine15. White arrows indicate micronuclei. OC47: highly differentiated serous cancer. **d**: Schematic representation of DNA metabolism in IR-induced MN that potentially perturbs genomic stability. Unrepaired DSBs in mitoses trigger MN formation in post-mitotic cells. Interphase MN continue to process DNA fragments into ssDNA. During next mitosis, MN disintegrate and release existing ssDNA that admix with chromosomal DNA and promote chromosomal shattering or high-frequent rearrangement in next cell cycle, causing illegitimate re-integration of DNA fragment into the genome

Apart from characterization of micronuclei formation in cell lines of ovarian cancer, we also monitored MN in biopsies of human ovarian cancer. Due to the compact volume of the cancerous cells and weak DAPI staining of micronuclei, we monitored DDR protein markers in off-nuclear compartments in tissues from clinical derived biopsies. In primary cultures of these tumor tissues, we visualized MN-like compartments by detecting cytoplasmic 53BP1 and γH2AX (fig. 5a and b). Clearly, large proportion of primary OC cells contained off-nuclear compartments with activated DDR signals. Mirroring the weak DAPI staining in late-stage MN of OVCAR-8 cells, many of the MN-like compartments in primary OC culture displayed invisible DAPI signal but easily detected γH2AX/53BP1, suggesting that DNA fragments was undergoing extensive structural changes. Moreover, similar to the sample of borderline stage (OC20, fig. 5a), immunostaining for phosphorylated TP53 in cryosections showed frequent spontaneous MN-like structures with positive pSerine15 in high-grade serous ovarian cancer samples (fig. 5c), indicating frequent emergence of MN during cancer development. Therefore, ovarian cancers from borderline to advanced stages are prone to develop micronuclei containing processed DNA structures that are potentially toxic to nuclear genomes.

## Discussion

DNA damage responses play important roles in maintaining genome stability. Unrepaired DNA lesions are transmitted to daughter cells, causing elevated levels of mutations, chromosomal aberration or tumor predisposition. Micronuclei are resulted from broken chromosomes, especially after chemo- or radio-therapies. Although the formation of micronuclei is traditionally considered as marker for genome instability, recent advances of genomic sequencing and the development of high-resolution microscopy have built up a more refined scenario of DNA damage and micronuclei [21]. DNA fragments in micronuclei is under replicated, insufficiently repaired, and when they are re-incorporated in the daughter nuclei, they contribute to genomic instability such as chromothripsis, a massive, complex and focal rearrangement restricted to only one or few chromosomes. Generation of micronuclei and ensuing massive rearrangement offers even better chance than point mutation for post-therapy cancer cells with respect of genomic variation and acquirement of potential of resistance and recurrence.

Previous in vitro MN assay showed two population of micronuclei: those with less than 1/4 the diameter of the main nuclei (Type I) and others with 1/4–1/2 of the size (Type II) [18]. Here we show that 53BP1 and γH2AX shown by immunostaining display strong co-localization in nuclei compartments but are segregated in MN, reflecting distinct kinetics and mechanism of DNA metabolism between the two compartments. Also, accumulation of ssDNA and RPA in late-stage MN is accordance to the disappearance of γH2AX foci and extensive processing of DSB ends indicated by diminished DAPI signals. Accordingly, micronuclei can be classified into two metabolism-related stages: early-stage compartments characterized with DAPI^strong^ γH2AX^+^ 53BP1^−^ ssDNA^−^ signals and compact size correspond to Type I MN; and those in late stages with DAPI^weak^ γH2AX^−^ 53BP1^+^ ssDNA+ are related to large volume Type II MN compartments. The early-stage MN represent those of recent mitotic exist, without extensive DNA end processing, whereas the late-stage counterparts contain extensively processed ssDNA.

It was proposed that DDR signaling activated by DSBs is incomplete in mitosis [27]. Thus, while signaling factors like γH2AX is recruited to damaged mitotic chromatin, other factors (53BP1) will be loaded after mitosis when the micronuclei have formed. In this regard, our observation of low ratio of overlapping γH2AX/53BP1 signal in micronuclei is consistent to previous report showing that not all MN display full co-localization between γH2AX and MRE11 or 53BP1 [28]. Although other reports explained that this lack of co-localization caused by cell cycle-specific recruitment of DDR factors and by nucleocytoplasmic transport defects of MN, our data also suggest the exclusion of γH2AX in late-stage MN might be caused by extensive processing of DNA.

To generate long stretches of ssDNA required for HR repair in nuclei, a series of nucleolytic enzymes are recruited to the DSBs. MRN and CTIP mediate the short-range resection while BLM-DNA2 carry out the long-range resection to produce functional RPA-coated ssDNA filaments. BRCA1 and 53BP1, as well as epigenetic factors like WDR70 that regulates the accessibility of repair factors in the vicinity of DSBs, constitute a tightly controlled resection promoter that determine the efficacy and extension of resection nucleases [13, 25].However, in the case of dicentric chromosome formed after telomere crisis, chromothripsis and kataegis requires cytoplasmic 3′-nuclease TREX1 to generate RPA-coated ssDNA and resolve chromatin bridges [29]. This could the case for micronuclei with membrane rupture that allows the access of cytoplasmic TREX1 to DNA fragments and generate ssDNA in MN. Thus, the TREX1-dependent 3′-nuclease activity may be a substitute mechanism for MRN-, BRCA1- and WDR70-independent resection in micronuclei.

The main obstacle for ovarian cancer therapy is the high incidence of recurrence and resistance. The first-line anti-OC therapy targeting DNA metabolism and chromosomal stability (Cisplatin and Taxol) cause genomic rearrangement and other mutagenic events, which are thought to be associated with quick development of drug resistance. Not until recent time, micronuclei is regarded as the results of the above genome instability mechanisms, but not the potent driving force of next round of genomic rearrangement. Because micronuclei can exist for 1–4 mitoses and the fates varies from exclusion from cells, re-integration into main nucleus, or being digested in situ [30], they can in turn trigger devastating genomic variation in next round of mitosis or upon spillage of MN contents (like ssDNA or other toxic structures). In accordance to this hypothesis, recent studies suggest that DSB-induced micronuclei cause large-scale genomic rearrangement including chromothripsis or chromosomal shattering, potentially contributes to quick changes of genomic variation in somatic genomes of tumor [31–33]. As such, the micronuclei-associated genomic evolution may provide alternative explanations to the resistance encountered in anti-tumor therapy. Indeed, our work strongly suggest that the presence of bulky amount of ssDNA in micronuclei can be potential substrates employed by illegitimate homologous recombination repair upon reintegrating into the nuclei compartments, causing catastrophic and localized rearrangement.

## Conclusions

Thus, our findings show the liability of MN-associated DNA to be processed into abnormal structures in ovarian cancer cells and offer a novel mechanism for the MN-driven genomic adaptation that eventually leads to drug resistance and recurrence.

## Abbreviations

CPT: Camptothecin
DAPI: 4′,6-diamidino-2-phenylindole
DDR: DNA damage responses
DSB: Double strand break
IR: Ionizing radiation
IRIF: Ionizing radiation-induced foci
MN: micronuclei
RPA: Replication protein A
ssDNA: Single –strand DNA
